# Empowering a Clinical Research Team to Recruit and Retain Hispanic and African American Participants for the DAWN Alzheimer's Research Study

**DOI:** 10.1002/alz70860_107293

**Published:** 2025-12-23

**Authors:** Takiyah D. Starks, Shawnta Lloyd, Kristy A. Terrell, Charles T Fennell, Lakisha D Hinnant, Ke'yona Barton, Rodney Witherspoon, Brianna Eley, Larry D Adams, Mary E Douglas, Tina Duke, Pedro R Mena, Katalina F. McInerney, Jose J. Sanchez, Vanessa C. Rodriguez, Glenies S. Valladares, Mariangelie Lopez, Katrina Celis, Jenny Chavez, Jennifer Cespedes, Renee A. Laux, Sarah Kennedy, Gabrielle Blackshire, Paris Pryor‐Banks, Allison M Caban‐Holt, Michael L Cuccaro, Jeffery M Vance, Jonathan L Haines, Giuseppe Tosto, Christiane Reitz, Rufus O Akinyemi, Margaret Pericak‐Vance, Goldie S Byrd

**Affiliations:** ^1^ Maya Angelou Center for Health Equity, Wake Forest University School of Medicine, Winston‐Salem, NC, USA; ^2^ Maya Angelou Center for Health Equity, Winston‐Salem, NC, USA; ^3^ Wake Forest University School of Medicine, Winston‐Salem, NC, USA; ^4^ Maya Angelou Center for Health Equity at Wake Forest University School of Medicine, Winston‐Salem, NC, USA; ^5^ John P. Hussman Institute for Human Genomics, University of Miami Miller School of Medicine, Miami, FL, USA; ^6^ John P. Hussman Institute for Human Genomics, Miami, FL, USA; ^7^ University of Miami Miller School of Medicine, Miami, FL, USA; ^8^ Columbia University Irving Medical Center, New York, NY, USA; ^9^ Gertrude H. Sergievsky Center, Taub Institute for Research on the Aging Brain, Departments of Neurology, Psychiatry, and Epidemiology, College of Physicians and Surgeons, Columbia University, New York, NY, USA; ^10^ Department of Population and Quantitative Health Sciences, Case Western Reserve University School of Medicine, Cleveland, OH, USA; ^11^ Case Western Reserve, Cleveland, OH, USA; ^12^ Department of Population and Quantitative Health Sciences, Case Western Reserve University, Cleveland, OH, USA; ^13^ Cleveland Institute for Computational Biology, Department of Population & Quantitative Health Sciences, School of Medicine, Case Western Reserve University, Cleveland, OH, USA; ^14^ College of Medicine, University of Ibadan, Ibadan, Oyo, Nigeria; ^15^ Dr. John T. Macdonald Foundation Department of Human Genetics, University of Miami Miller School of Medicine, Miami, FL, USA

## Abstract

**Background:**

The DAWN study will build a large genomics resource for AD research in individuals with African (Af) and Hispanic/Latino (H/L) ancestry. Clinical Research Coordinators (CRCs) are recruiting 5,000 Af, 4,000 African Americans (AA) and 4,000 H/L participants from the US and ten African sites. Study recruitment is done through community engagement in rural and urban settings. Coordinators on the front lines play an invaluable role in the success of reaching DAWN goals and assuring participant satisfaction as well as study trustworthiness. Often too little investment is made in this indispensable group.

**Method:**

DAWN utilizes a team science approach for recruiting AA and HI participants across four U.S. sites. Outreach and Recruitment (OR) efforts are led by an OR core team. Clinical trainings are led by a Coordinating Site team and by managers at each site. Weekly virtual meetings hosted by the Coordinating Site, and monthly collaborators meetings offer bi‐directional learning between CRCs and trainers. An annual CRC meeting creates platforms for advancing professional development, clinical skills, culturally relevant and patient‐centered recruitment strategies, and camaraderie among cross‐site peers. At face‐to‐face convenings teams review study goals and expectations, celebrate milestones, and empower CRCs to identify organizational, administrative and personal challenges and successes across sites, including collaborating across‐sites to achieve enrollment goals.

**Result:**

Nineteen DAWN CRCs are of AA and HI descent, representative of the recruitment populations. Approximately, 85% are female and all have at least a bachelor's degree. In the first two years, DAWN CRCs met enrollment goals with (1,616 AAs and 1,599 HIs). They identified 26 impediments and 14 retention strategies to improve recruitment efficiencies. Top strategies involved building community trust, CRC safety, providing additional in‐person trainings and providing resources for community. CRCs at all DAWN sites have assisted at least one other site in their recruitment.

**Conclusion:**

Multi‐site efforts to provide important ancestral diversity to the global Alzheimer's genomics research effort will continue needing to identify roles, impediments and strategies for successful recruitment and retention of participants. Listening to the voices of CRCs and investing in their development are important strategies for recruitment success and employee retention.

